# Flavonoids of *Polygonum hydropiper L*. attenuates lipopolysaccharide-induced inflammatory injury via suppressing phosphorylation in MAPKs pathways

**DOI:** 10.1186/s12906-016-1001-8

**Published:** 2016-01-22

**Authors:** Junyu Tao, Yingyi Wei, Tingjun Hu

**Affiliations:** College of Animal Science and Technology, Guangxi University, Nanning, 530005 P.R. China

**Keywords:** Flavonoids, Antioxidant activity, Anti-inflammatory, AMPK, MAPKs, Phosphorylation

## Abstract

**Background:**

*Polygonum hydropiper L.* is widely used as a traditional remedy for the treatment of dysentery, gastroenteritis. It has been used to relieve swelling and pain, dispel wind and remove dampness, eliminate abundant phlegm and inflammatory for a long time. Previous study showed that antioxidants especially flavonoids pretreatment alleviated sepsis-induced injury in vitro and in vivo. In the present study, the possible anti-inflammatory effect of flavonoids from normal butanol fraction of *Polygonum hydropiper L.* extract (FNP) against inflammation induced by lipopolysaccharide (LPS) was evaluated in vivo and in vitro.

**Methods:**

The content of total flavonoid of FNP was determined by the aluminum colorimetric method. The content of rutin, quercetin and quercitrin was determined by HPLC method. Mice received FNP orally 3 days before an intra-peritoneal (i.p.) injection of lipopolysaccharide (LPS). Total superoxidase dismutase (T-SOD), total antioxidant capacity (T-AOC), glutathione peroxidase (GSH-PX), glutathione (GSH), myeloperoxidase (MPO) and malondialdehyde (MDA) levels were measured. Tumor necrosis factor-α levels in serum and tissue was measured. mRNA expressions of pro-inflammatory cytokines in lung were assessed by Real-Time PCR. Histopathological changes were evaluated in lung, ileum and colon. We also investigated FNP on reactive oxygen species (ROS), nitric oxide (NO) and pro-inflammatory cytokines (TNF-α, IL-1β, IL-6 and IL-8) production, inducible nitric oxide synthase (iNOS), Cyclooxygenase-2 (COX-2) protein expression, phosphorylation of MAPKs and AMPK in LPS-stimulated RAW264.7 cells.

**Results:**

FNP increased the levels of T-SOD, T-AOC, GSH-PX and GSH, decreased the levels of TNF-α, MPO and MDA, attenuate the histopathological lesion in LPS-stimulated mice. FNP inhibited production of inflammatory cytokines, ROS and NO, protein expressions of iNOS and COX-2, phosphorylation of ERK, JNK and c-JUN in MAPKs, promoted phosphorylation of AMPKα suppressed by LPS.

**Conclusion:**

These results suggested in vivo anti-inflammatory activities of FNP might contributed to its enhancement in antioxidant capacity, its inhibitory effects may be mediated by inhibiting the phosphorylation of JNK, ERK and c-JUN in MAPKs signaling pathways.

## Background

Sepsis, a systemic inflammatory response syndrome induced by infection [1], is the most common cause of death in intensive care units [2–4]. It can be defined as a systemic inflammatory disorder and characterized clinically by fever [5], enteritis [6] and acute pneumonia [7]. Previous studies have established that dysfunction of neutrophils granulocyte [8], microcirculation disorder [9], mitochondria dysfunction [10] and translocation of endotoxin [11] are involved in the sepsis course. To investigate the underlying mechanisms of clinical sepsis shock and develop an effective therapeutic strategies to endotoxin challenge, bacterial LPS, the outer membrane component of gram-negative bacterial cell walls [12], is widely used to induce experimental endotoxemia in laboratory animals. Antioxidants alleviated sepsis-induced organ injury. In septic rats and pigs, treatment with N- acetylcysteine did protect rats and pigs against oxidative stress and improves survival rate [13, 14]. Ferulic acid, a well-established natural antioxidant, increased the GSH levels and SOD and GSH-PX activities, decreased the MDA levels and DNA damage in the sepsis-induced rats [15]. Therefore, agents with antioxidant activity that suppress the production of inflammatory cytokines and inflammatory mediators may have therapeutic effects.

Macrophages are mainly responsible for initiating the immune defense and inflammatory reactions. Previous research indicated that LPS-induced iNOS and COX-2 expression in macrophages are regulated by mitogen-activated protein kinases (MAPKs), including extracellular signal regulated kinase (ERK), c-Jun N terminal kinase (JNK), and p38 [16]. Among them, JNK MAPKs pathway is crucial for macrophage activation and the production of inflammatory cytokines [17]. A wide variety of flavonoid molecules possess anti-inflammatory activity in LPS-induced macrophage by regulating MAPKs signaling pathway [18–20]. Fenoterol inhibits LPS-induced AMPK activation and IL-1β production, AMPKα1 subunit contributed to LPS-induced release of pro-inflammatory cytokines in THP-1 cells [21]. Therefore, treatments aimed at regulating AMPK and MAPKs may have potential therapeutic advantages for inflammatory diseases.

The folk medicinal plant, *Polygonum hydropiper L*. which is widely used in East Asian countries for centuries, especially in Korea, Japan and China. Flavonoids isolated from methanol extract of *Polygonum hydropiper L*. leaves showed strong antioxidant activity [22, 23]. Quercetin was found as one of the active ingredients in 99 % methanol extracts of this plant to inhibits NO and PGE2 production in LPS-treated macrophages [24]. Considering the antioxidant activity and anti-Inflammatory activity of the *Polygonum hydropiper L.*, it is surprising that no pharmacological study has been performed for investigating the protective effect on LPS-induced sepsis in vivo. We hypothesize that flavonoids of *Polygonum hydropiper L.* might has an anti-inflammatory activity. However, there is no scientific evidence, which validate its use in the literature. Therefore, the aim of the present study is to evaluate whether the flavonoids of normal butanol extract from *Polygonum hydropiper L.*have actions on the production of inflammatory mediators from macrophage so as to inhibit inflammation and protect mice against LPS-induced sepsis.

## Methods

### Materials and reagents

LPS (*Escherichia coli* 055:B5) was purchased from Sigma Chemical Co. (St. Louis, USA). Foetal bovine serum and Dulbecco Modified Eagle Mediumas (DMEM) was purchased from Gibco (New York, USA). 3-(4, 5-Dimethylthiazol-2-yl)-2, 5-diphenyltetrazolium bromide (MTT) and Fluorescent probe dichlorofluorescin diacetate (DCFH-DA) for ROS dection were purchased from Applygen (Beijing, PR China). Mouse TNF-α, IL-6, IL-8, IL-10 and IL-1β enzyme-linked immuno sorbent assay (ELISA) kits were purchased from Neobioscience (Shenzhen, PR China). RAW264.7 cells were purchased from the Type Culture Collection of Chinese Academy of Sciences (Shanghai, PR China). Assay kits for NO, T-SOD, T-AOC, GSH-PX, GSH, lysozyme (LZM), acid phosphatase (ACP), MPO, MDA dection were purchased from the Institute of Nanjing Jiancheng Bioengineering (Nanjing, PR China). RNAiso Plus Kit, PrimeScript® RT reagent Kit (Perfect Real Time) and SYBR® Premix Ex TaqTM II Kit (Perfect Real Time) used in RT-qPCR were purchased from Takara Biotechnology (Dalian, PR China). Cell culture dishes and plates (12 mm polycarbonate membrane, 0.4 μm pore size, 1.12 cm_2_ surface area) were purchased from Corning (New York, USA). iNOS (D6B6S), COX-2 (D5H5), MAPKs Family Antibody Sampler Kit, phospho-MAPKs Family Antibody Sampler Kit, c-JUN (60A8), phospho-c-JUN (Ser 3), AMPKα (D5A2), phospho-AMPKα (40H9), β-Actin (13E5) and GADPH (14C10) were obtained from Cell Signaling Technology (Beverly, USA).

### Herbal extract preparation

*Polygonum hydropiper L*. was purchased from Tai Hua pharmaceutical Co. Ltd. The plant material was authenticated by Professor Renbin Huang, School of Pharmacy, Guangxi medical University. The voucher specimen (2010–125) has been deposited at the Herbarium of Faculty of Pharmacy, Guangxi medical University for future reference. The extraction and purification of FNP were carried out as followed. Briefly, the air-dried whole plant (500 g) was ground to a powder and extracted with ultrasonic in a buffered solution(Hac-NaAc pH 4.8,1.5 l) added with cellulase and pectinase (0.25 % W/V respectively) at 50 °C for 1.5 h. Then the pH value was adjusted to 9.0 with sodium carbonate solution and the enzyme was inactivated at 85 °C water bath for 15 min, and then macerated with ethanol for 24 h. The extracts were filtered and residue were extracted by maceration in ethanol. The combined filtrate was evaporated under reduced pressure to obtain a crude extract (yield 52.3 %) and successively treated with petroleum ether (40–60 °C), chloroform, ethyl acetate and normal butanol. The normal butanol fraction yield was 11.96 %. The normal butanol fraction was added into column loading-treated XDA-8 macroreticular resin for adsorption for 24 h, and then washed with water and 20 % ethanol, respectively, to get rid of polar impurities. FNP in the column was eluted with 75 % ethanol, and the eluting solution was dried in vacuum condition until yellow powders were achieved.

### Quality control of FNP

Approximately 100 mg of FNP were transferred to a 10 mL volumetric flask and diluted with methanol to volume. The test sample was finally filtered through a 0.45 μm membrane filter before analysis. The total flavonoids content was determined using a colorimetric method as previously described but with slightly modified [25]. Briefly, 1.0 mL of NaNO_2_ solution (5 %, w/v), 1.0 mL of AlCl_3_ solution (10 %), and 4.0 mL NaOH solution (1.0 mol/L) were mixed with the same volume of the test sample. The final volume was adjusted to 25 mL with methanol (80 %, v/v). The mixture was allowed to stand for 5 min and the absorbance was measured at 510 nm against the same mixture, without the sample as a blank. Rutin was used as a reference standard and the total flavonoids content was expressed as rutin. The calibration curve (Y = 0.0011X + 0.00125 where X was absorbance value of sample, and Y was sample concentration) was ranged from 100 to 500 μg/mL (R_2_ = 0.9992). The contents of three markers (rutin, quercetin and quercitrin) in the extract samples were determined by High Performance Liquid Chromatography (HPLC) using followed chromatographic condition: Waters e2695 separation module and Waters 2998 photodiode array detector were controlled by EMPOWER™ 2 chromatography data sofeware (Waters Co. Ltd. USA). Waters X Bridge™ C18 HPLC column (4.6 × 250 mm 5 μm, Waters Co. Ltd. USA). Column temperature was kept constantly at 30°C. UV detection wavelength was 254 nm. The injection volume was 10 μL. The isocratic mobile phase consisted of acetonitrile-0.3 % phosphoricacid (27:73, v/v) was used to elute for 40 min and the flow rate was kept at 1.0 mL/min.

### Animals

The study protocol was approved by the Ethics Committee of Guangxi University and was performed in accordance with the Guiding Principles for the Care and Use of Laboratory Animals. Male special pathogen free KM mice were obtained from the Laboratory Animals Center of Guangxi Medical University (Certificate No. 2011–002). After three days of acclimatization, 50 mice were randomly divided into five groups of 10 mice each. Mice in the LPS alone group were injected intraperitoneally with 17 mg/kg (body weight) of Escherichia coli LPS (Serotype 055:B5, Sigma) in phosphate-buffered saline (PBS) [26], while control mice were injected with only PBS (10 mL/kg). The animals in FNP50, FNP100 and FNP200 group were orally administrated with diverse dose FNP (50 mg/kg, 100 mg/kg or 200 mg/kg) for 3 days before instillation of LPS. All mice were euthanized at 24 h after LPS or PBS instillation, and the samples were collected for subsequent analysis.

### Mortality and measurement of TNF-α values

The mortality of mice was recorded for 24 h after the LPS challenge in each treated group. Homogenates of murine liver and intestine were prepared using physiological saline (1:9, w/v ratio) and kept on an ice bath. The homogenates were then centrifuged at 1,000 g at 4 °C for 15 min, and the supernates were collected. Blood samples were collected and the TNF-α levels in serum and supernates was analyzed using a cytokine-specific ELISA kit according to the manufacturer’s protocol.

### Biochemical analysis

Homogenates of murine small intestine were prepared using physiological saline (1:9, w/v ratio) and kept on an ice bath. The homogenates were then centrifuged at 1,000 g at 4 °C for 15 min, and the supernates were collected and analyzed for MPO activities and MDA levels. Homogenates of murine liver were prepared using physiological saline (1:9, w/v) and kept on an ice bath. The homogenates were then centrifuged at 1,000 g at 4 °C for 15 min, and the supernates were collected and analyzed for T-SOD, T-AOC and GSH-PX activities. Blood samples were collected and the serum were then centrifuged at 1,000 g at 4 °C for 15 min, and the supernates were collected and analyzed for levels of LZM, ACP and GSH.

### Quantification of messenger RNA (mRNA) in lung of mice

Total RNA was extracted from lung tissues by using RNAiso Plus Kit, and RNA was reverse transcribed to cDNA using reverse transcription and amplified by PCR with PrimeScript® RT reagent Kit according to the manufacturer’s instructions. Relative expression of TNF-α、IFN-α、IFN-γ and IL-2 were measured with a SYBER green detection system by using CFX96 Real-Time PCR Detection System (Bio-Rad, USA), Each reaction was run in triplicate and the cycle threshold (CT) values for each mRNA were subtracted from that of β-actin mRNA averaged and converted from log-linear to linear term. The primer sequences used were as follows: TNF-α (NM_013693): forward: 5'-AAGACCTCTATGCCAACACAGT-3' and reverse: 5'-TTTACTCAGTGCCAGAAGCTGGA-3’; IL-2 (NM_008366.3): forward: 5'-CCCAGGATGCTCACCTTCA-3' and reverse: 5'-CCGCAGAGGTCCAAGTTCA-3’; IFN-α (NM_010502.2): forward: 5'-CTGTGCTTTCCTGATGGTCCTG-3’ and reverse: 5'-GGAATCCAAAGTCCTTCCTGTCCT-3'; IFN-γ (NM_00801778): forward: 5'-GCTTTGCAGCTCTTCCTCATG-3' and reverse: 5'-CTTCCACATCTATGCCACTTGAG-3'; β-actin (NM_007393.3): forward: 5'-AAGACCTCTATGCCAACACAGT-3' and reverse: 5'-TTTACTCAGTGCCAGAAGCTGGA-3'.

### Histopathology

To characterize the histological alterations, the lungs, ileum and colon of the mice were excised and fixed in 10 % neutral buffered formalin. The tissue samples were dehydrated with graded alcohol, embedded in paraffin, and the sections stained with hematoxylin and eosin were examined by light microscopy.

### Cell culture and treatments

Mouse macrophage RAW 264.7 cells were cultured in DMEM supplemented with 10 % heat-inactivated fetal bovine serum,100 units/mL penicillin sodium, 100 μg/mL strepto-mycin and 2 mmol/L glutamine at 37 °C under a humidified atmosphere of 5 % CO_2_. In all experiments, cells were allowed to acclimate for 24 h before any treatment. Cells were incubated with or without FNP that was added 1 h prior to LPS treatment.

### MTT assay for testing cell viability

The examination of cytotoxicity induced by FNP was performed by MTT assay [27]. RAW 264.7 cells were mechanically scraped, plated at a density of 2 × 10^5^ cells /mL into 96-well plates and incubated in a 37 °C, 5 % CO_2_ incubator overnight. FNP was dissolved in Dimethyl Sulphoxide (DMSO), and the DMSO concentrations in all assays was not exceed 0.1 %. After overnight incubation, the cells were treated with diverse concentrations of FNP (20–400 μg/mL) in the presence or absence of LPS (1 μg/mL) according to the experimental design. After 20 h, 20 μL of 5 mg/mL MTT was added to each well and the cells were further incubated for an additional 4 h. MTT was removed and cells were lysed with 150 μL/well DMSO. The optical density was measured at 570 nm on a microplate reader.

### Measurement of NO production

After pre-incubation of RAW264.7 cells (1 × 10^6^ cells/mL) for 18 h, cells were pre-treated with FNP (20–80 μg/mL) for 1 h and were further incubated with LPS (1 μg/mL) for 12 h or 24 h. The inhibitory effect of FNP on NO production was determined by Griess reagent as described previously [28].

### Measurement of ROS levels

After pre-incubation of RAW264.7 cells (1 × 10^6^ cells /mL) for 18 h, the cells were pre-treated with FNP (20, 40 or 80 μg/mL) for 1 h and were further incubated with LPS (1 μg/mL) for 12 h or 24 h. The inhibitory effect of FNP on ROS production was determined by fluorescent probe DCFH-DA as described previously [29].

### Measurement of inflammatory cytokine values

To investigate the effect of FNP on inflammatory cytokine levels from LPS-treated RAW 264.7 cells, The pre-incubated cells (1 × 10^6^ cells /mL) were pretreated with 20, 40 or 80 μg/mL of FNP for 1 h prior to 1 h treatment with 1 μg/mL LPS at 37 °C, 5 % CO_2_ incubator. 24h later post LPS treatment, cell-free supernatants were collected and stored at −20 °C until assayed for cytokine levels. The concentrations of TNF-α, IL-6, IL-8, IL-10 and IL-1β in the supernatants of RAW 264.7 cell culture were determined by a cytokine-specific ELISA kit, according to the manufacturer’s instructions.

### Western blot analysis

RAW 264.7 cells (2 × 10^5^ cells/mL) were plated in 24-well plates and pretreated with 20 μg/ mL or 80 μg/ mL of FNP for 2 h or 4 h and then stimulated with 1 μg/mL of LPS for 1 h. After incubation, the cells were collected and washed twice with cold PBS. The washed cell pellets were resuspended in extraction lysis buffer (1:10 10 × RIPA Buffer(CST,USA),1:10 cOmplete ULTRA EDTA-free protease inhibitor cocktail tablets (Roche, Swiss), 1:10 PhosSTOP Phosphatase Inhibitor Cocktail Tablets (Roche, Swiss)) and maintained on ice for 30 min. The lysates were centrifuged (12,000 g at 4 °C) for 5 min to obtain the cytosolic fraction and the protein concentrations were determined using a BCA™ protein assay kit (Cwbiotech, China) according to the manufacturer’s instructions. Aliquots of the cell extracts (50 μg of protein) were separated on 10 % SDS-polyacrylamide gel and transferred onto a polyvinylidene fluoride (PVDF) membrane (Roche, Swiss) with a glycine transfer buffer [192 mM glycine, 25 mM Tris–HCl (pH 8.8), 10 % methanol (v/v)]. The membrane was incubated overnight with specific primary antibody(CST,USA) at 4 °C after blocking the nonspecific site with 5 % bovine serum albumin (BSA). The membrane were washed with Tween 20/Tris buffered saline [TTBS, 20 mM Tris–HCl buffer, pH 7.6, containing 137 mM NaCl and 0.05 % (v/v) Tween 20] and incubated an additional 60 min with a peroxidase-conjugated secondary anti-mouse antibody(1:5000 CST,USA) at room temperature. The membranes were washed with TTBS twice and the immunoactive proteins were detected using an enhanced chemiluminescence (ECL) Western blotting detection kit (Merck Millipore, Germany).

### Statistical analysis

All experimental data in this study was represented as mean ± SD. All statistical comparisons were conducted using oneway ANOVA followed by Tukey’s test and analyzed using SPSS 16.0 for Windows. Difference was considered statistically significant when *P* < 0.05, whereas less than 0.01 were considered extremely significant (*P* < 0.01).

## Results

### Quality evaluation of FNP

**Fig. 1:**
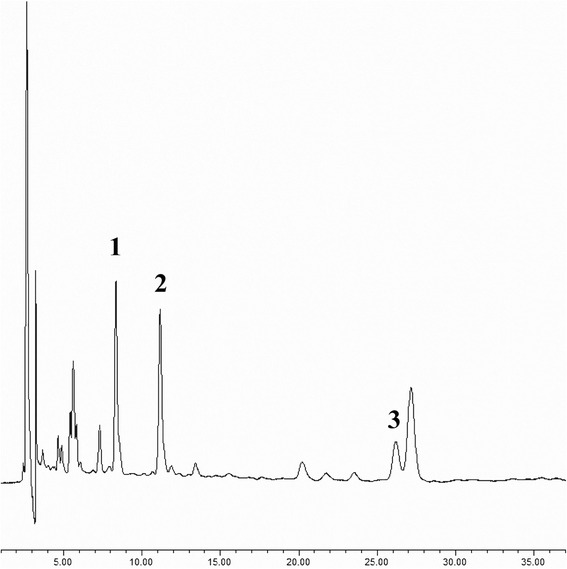
HPLC analysis of FNP. Notes: The isolated compounds were identified in the extract by comparing their retention times. The chromatograms were obtained at a wavelength of 254nm. (1) rutin (2) quercitrin (3) quercetin

The content of total flavonoids in the extract was determined quantitatively as 55.3 % by colorimetric method by using rutin as the standard. The contents of rutin, quercetin and quercitrin in the extract detected by HPLC analysis (Fig. 1) is 21.9 %, 8.2 % and 20.6 %, respectively.

### FNP reduced mortality and pathological damages in LPS-stimulated mice

**Fig. 2:**
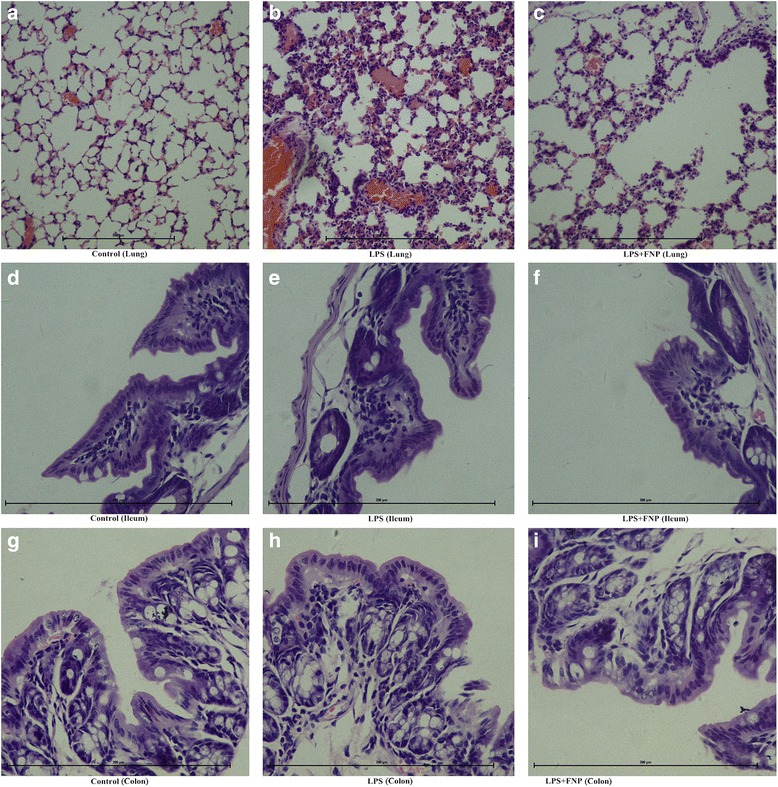
Protective effect of FNP on lung, ileum and colon in LPS-stimulated mice. Notes: The mice in the LPS group were injected intraperitoneally with 17 mg/kg (body weight) of LPS, while control group were injected with PBS (10 mL/kg). FNP50, FNP100 and FNP200 group were orally administrated with diverse dose FNP (50 mg/kg, 100 mg/kg and 200 mg/kg) for 3 days before instillation of LPS. Histopathological studies by light microscope showing morphologically normal lung, ileum and colon tissues from mice in the control group (**a**, **d** and **g**). Incrassation of alveolar wall, serous exudation, hemorrhage and infiltration of PMN in lung from LPS-treated group (**b**) and no serous exudation or hemorrhage and less incrassation of alveolar wall and infiltration by PMN in lung from LPS-treated mice treated with FNP (**c**). Necrosis of epithelial cell, disorder of epithelial cell arrangement and fall of epithelium of intestinal villus in ileum and colon from LPS-treated group (**e** and **h**) and less necrosis of epithelial cell in ileum and colon from LPS-treated mice treated with FNP (**f** and **i**). Tissue sections were stained with hematoxylin and eosin and view by light microscopy (200 and 400×)

First, we examined the effect of FNP (50, 100 or 200 mg/kg oral administration) on LPS (17 mg/kg, intraperitoneally) induced mortality for 24 h in mice. No mice were dead in control group while LPS treated alone caused 30 % mortality within 24 h. Pretreated with FNP significantly reduced LPS induced death. No mice were dead while pretreated with FNP. As shown in Fig. 2, no obvious histopathological change was found in lung from control group (Fig. 2a). Remarkable incrassation of alveolar wall, serous exudation, hemorrhage and infiltration of polymorphonuclear neutrophil (PMN) was presented in LPS-treated group (Fig. 2b). No serous exudation and hemorrhage, less incrassation of alveolar wall and less infiltration by PMN was shown in FNP pretreated groups (Fig. 2c) as compared with LPS-treated group. No obvious histopathological change was found in ileum from control group (Fig. 2d). Necrosis of epithelial cell, disorder of epithelial cell arrangement and fall of epithelium of intestinal villus was presented in LPS-treated group (Fig. 2e). Less epithelial cell necrosis and less fall of epithelium of intestinal villus was shown in FNP pretreated groups (Fig. 2f) as compared with LPS-treated group. No obvious histopathological change was found in colon from control group (Fig. 2g). Indistinct villus border, necrosis of epithelial cell, disorder of epithelial cell arrangement and fall of epithelium of intestinal villus was presented in LPS-treated group (Fig. 2h). Less epithelial cell necrosis and less fall of epithelium of intestinal villus was shown in FNP pretreated groups (Fig. 2i) as compared with LPS-treated group.

### FNP reduced TNF-α levels in blood and tissues in LPS-stimulated mice

**Fig. 3:**
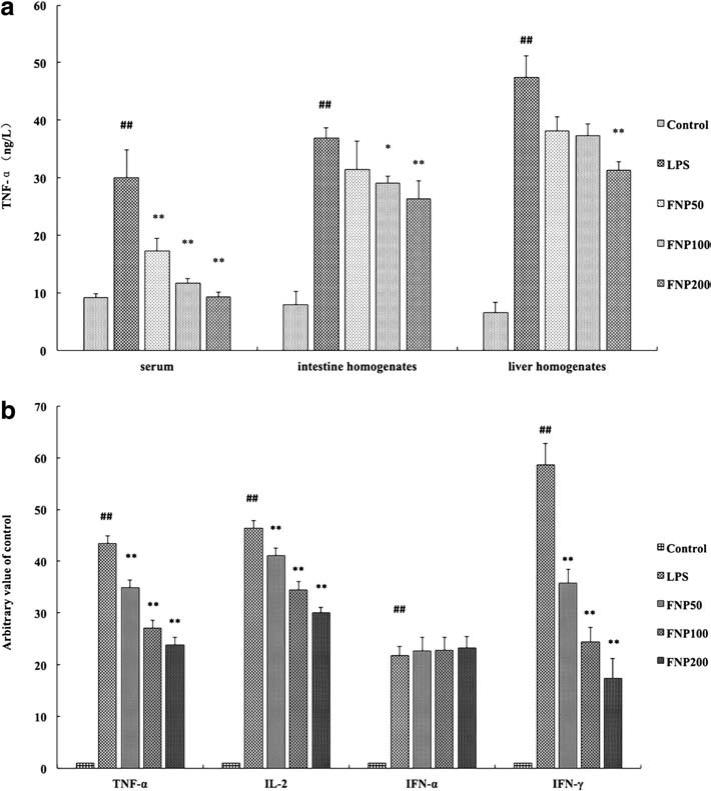
Effects of FNP pretreatment on TNF-α levels in blood and tissues, mRNA expression in lung. Notes: The mice in the LPS group were injected intraperitoneally with 17 mg/kg (body weight) of LPS, while control group were injected with PBS (10 mL/kg). FNP50, FNP100 and FNP200 group were orally administrated with diverse dose FNP (50 mg/kg, 100 mg/kg and 200 mg/kg) for 3 days before instillation of LPS. Serum and lung were collected, liver and intestine homogenates were prepared. TNF-α levels of serum and homogenates were shown in (**a**). The level of mRNA expression in lung is expressed as fold-change relative to the control group (**b**). The mRNA expression of TNF-α, IFN-α, IFN-γ and IL-2 was analyzed by qRT-PCR. Data are presented as the means ± SD from three independent experiments (*n* = 10). _##_ *P* <0.01 vs control group; _*_ *P* <0.05 vs LPS group; _**_ *P* <0.01 vs LPS group

As shown in Fig. 3a, mice with LPS treated alone resulted in significant increases in TNF-α production in serum, liver and intestine homogenates as compared to the control group. The production of TNF-α was 29.9 ± 4.8, 36.9 ± 3.7, 47.5 ± 3.6 ng/L respectively. However, the production of TNF-α in serum, liver and intestine homogenates was significantly inhibited as pretreated with FNP as compared with LPS-treated group. FNP pretreatment (200 mg/kg) reduced this TNF-α level by 68.7 %, 28.5 % and 34.1 % respectively. These results show FNP reduced TNF-α levels on LPS-stimulated mice.

### FNP down regulated mRNA expression in lung in LPS-stimulated mice

The effects of FNP on TNF-α, IFN-α, IFN-γ, IL-2 and IL-10 mRNA expressions were detected by qRT-PCR. As shown in Fig. 3b, mice with LPS treated alone resulted in significant increases in TNF-α (43.4 ± 1.5), IFN-α (21.8 ± 1.8), IFN-γ (58.6 ± 4.2) and IL-2 (46.4 ± 1.5) mRNA expression levels in lung as compared to the control group. However, pretreated with FNP (50, 100 or 200 mg/kg) significantly decreased mRNA expression level of TNF-α, IFN-γ and IL-2 as compared to the LPS-treated group. FNP did not inhibited mRNA expressions of IFN-α induced by LPS.

### FNP increased antioxidant capacity in LPS-stimulated mice

**Fig. 4:**
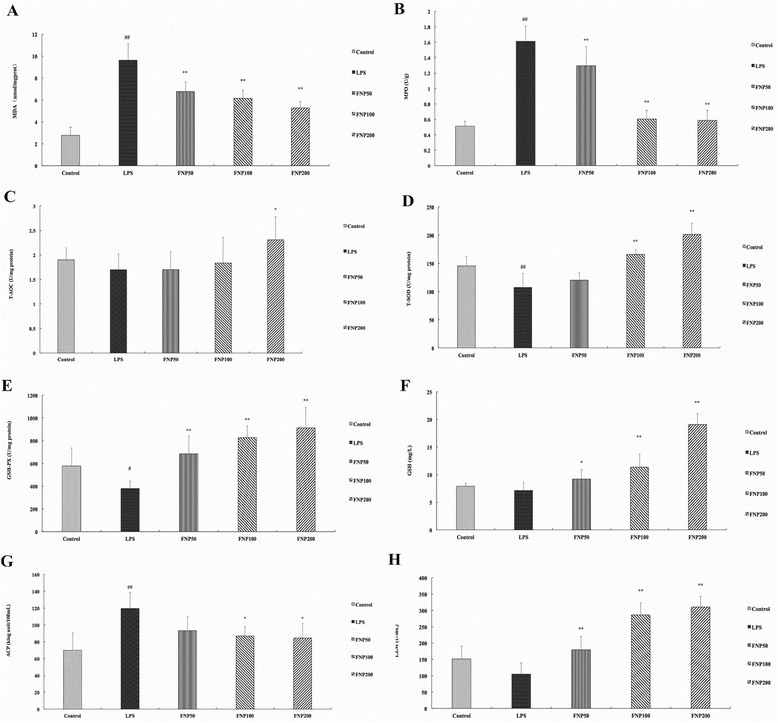
Effects of FNP on antioxidant capacity in LPS-stimulated mice. Notes: The mice in the LPS group were injected intraperitoneally with 17 mg/kg (body weight) of LPS, while control group were injected with PBS (10 mL/kg). FNP50, FNP100 and FNP200 group were orally administrated with diverse dose FNP (50 mg/kg, 100 mg/kg and 200 mg/kg) for 3 days before instillation of LPS. Serum, homogenates of small intestine and liver were prepared. (**a**) MDA level in small intestine. (**b**) MPO level in small intestine. (**c**) T-AOC level in liver. (**d**) T-SOD level in liver. (**e**) GSH-PX level in liver. (**f**) GSH level in serum. (**g**) ACP level in serum. (**h**) LZM level in serum. Each column represented as the means ± SD from three independent experiments (*n* = 10). _##_ *P* <0.01 vs control group; _*_ *P* <0.05 vs LPS group; _**_ *P* <0.01 vs LPS group

As shown in Fig. 4a and 4b, mice with LPS treated alone resulted in significant increases in MDA (9.6 ± 1.5 nmol/mgprot) and MPO (1.6 ± 0.2 U/g) production in small intestine homogenates as compared to the control group. The MDA and MPO level was increased by 247.6 % and 216.1 % as compared to the control group respectively. However, the production of MDA in small intestine homogenates was significantly inhibited as pretreated with FNP (50, 100 and 200 mg/kg) as compared with LPS-treated group, the MDA level was decreased by 29.6 %, 36.1 % and 45.1 % respectively. Pretreated with FNP (50, 100 and 200 mg/kg) significantly inhibited the increase of MPO in small intestine homogenates as compared with LPS-treated group, the MPO level was decreased by 19.6 %, 62.5 % and 63.7 % respectively.

As shown in Fig. 4c, no significant difference but a decrease was shown in T-AOC level between LPS-treated group and control group. However, the level of T-AOC in liver homogenates was significantly promoted as pretreated with FNP at the dose of 200 mg/kg as compared with LPS-treated group, the T-AOC level was increased by 36.1 %. As shown in Fig. 4d, mice with LPS treatment alone resulted in significant decreases in T-SOD (107.3 ± 24.7 U/mgprot) activity in liver homogenates as compared to the control group, the T-SOD level was decreased by 26.2 %. However, pretreated with FNP (100 and 200 mg/kg) significant increases T-SOD activities as compared to the LPS-treated group, the T-SOD level was increased by 54.6 % and 87.2 % respectively. As shown in Fig. 4e, mice with LPS treatment alone resulted in significant decreases in GSH-PX (379.7 ± 64.8 U/mgprot) activities in liver homogenates as compared to the control group, the GSH-PX level was decreased by 34.2 %. However, pretreated with FNP (50, 100 and 200 mg/kg) significant increases GSH-PX level as compared to the LPS-treated group, the GSH-PX level was increased by 80.3 %, 117.5 % and 140.3 % respectively.

As shown in Fig. 4f, no significant difference but a decrease was shown in GSH level between LPS group and control group. However, the level of GSH in serum was significantly promoted as pretreated with FNP (50, 100 and 200 mg/kg) as compared with LPS-treated group, the GSH level was increased by 29.2 %, 59.3 % and 168.0 % respectively. As shown in Fig. 4g, mice with LPS treatment alone resulted in significant increases in ACP (119.5 ± 19.3 king unit/100 mL) level in serum as compared to the control group, the ACP level was increased by 70.9 %. However, pretreated with FNP (100 and 200 mg/kg) significantly decreased ACP level as compared to the LPS-treated group, the ACP level was decreased by 27.4 % and 29.3 % respectively. As shown in Fig. 4h, no significant difference but a decrease was shown in LZM level between LPS group and control group. However, pretreated with FNP (50, 100 and 200 mg/kg) significantly increased LZM level as compared to the LPS-treated group, the LZM level was increased by 71.0 %, 172.5 % and 195.4 % respectively.

### Effects of FNP on RAW 264.7 macrophage viability

**Fig. 5:**
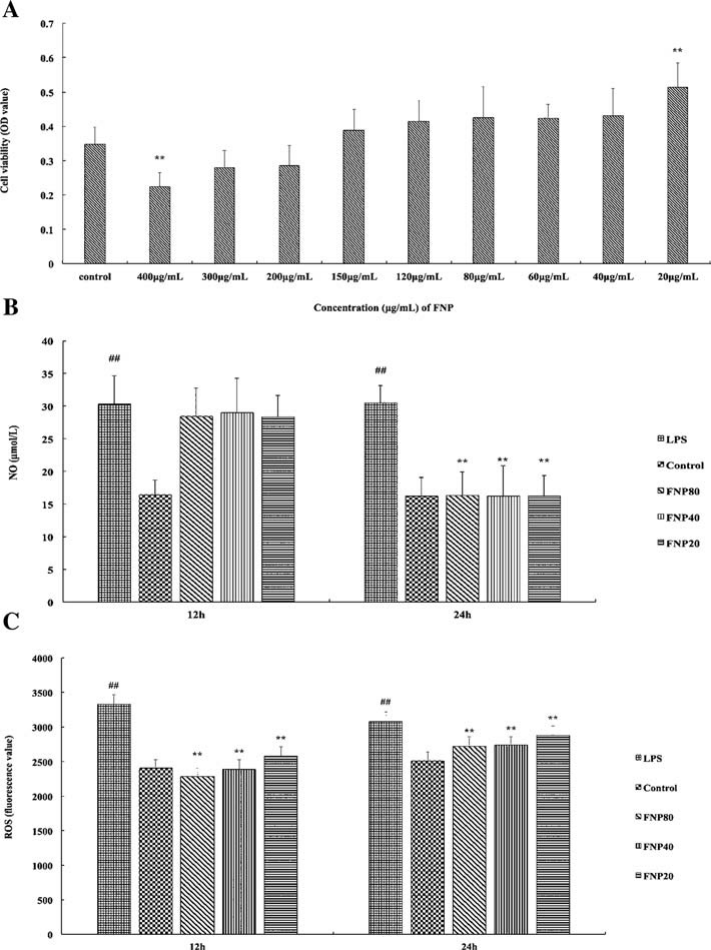
Cytotoxic effects of FNP in RAW264.7 cells (**a**) and the effects of FNP on LPS-induced NO (**b**), and ROS (**c**) productions in RAW264.7 macrophages. Notes: The cells were incubated for 24 h with 1 μg/mL of LPS in the absence or presence of FNP (20, 40, and 80 μg/mL). FNP was added 1 h before incubation with LPS. Cell viability assay was performed by using MTT assay. Nitrite concentration in the medium was determined by using Griess reagent. ROS production was determined by fluorescent probe DCFH-DA. Each column represented as the means ± SD from three independent experiments. _##_ *P* <0.01 vs control group; _*_ *P* <0.05 vs LPS group; _**_ *P* <0.01 vs LPS group

To assess the suitable concentration of FNP for the experiment, RAW 264.7 cells were incubated with FNP at concentrations ranging from 20 to 400 μg/mL and cell viability was measured by MTT test 24 h later. FNP at concentrations from 20 to 300 μg/mL had no cytotoxic effect on RAW 264.7 cells. An extremely higher cell viability was observed in the cells treated with FNP at concentration 20 μg/ml compared to the control group (*P* < 0.01) (Fig. 5a), therefore, we chose 40 and 80 μg/mL to investigate intervention effects of FNP in our further experiment.

### FNP reduced NO and ROS production in LPS-stimulated RAW 264.7 macrophages

Nitrite concentration in the medium was determined by using Griess reagent. ROS production was determined by fluorescent probe DCFH-DA. RAW 264.7 cells with LPS treated alone resulted in extremely significant increases in NO production as compared to the control group. The NO level was increased by 87.4 % as compared to control group. However, pretreated with FNP (80, 40 and 20 μg/mL) significantly inhibited the production of NO in 24 h after LPS stimulated (Fig. 5b). The NO level was decreased by 46.5 %, 46.9 % and 46.9 % respectively. RAW 264.7 cells with LPS treated alone resulted in significant increases in ROS production as compared to the control group. The ROS level was increased by 38.5 % as compared to control group. However, the production of ROS was significantly inhibited as pretreated with FNP (80, 40 or 20 μg/mL) 12 h and 24 h after LPS stimulated (Fig. 5c).

### FNP reduced inflammatory cytokine production in LPS-stimulated RAW 264.7 macrophages

**Fig. 6:**
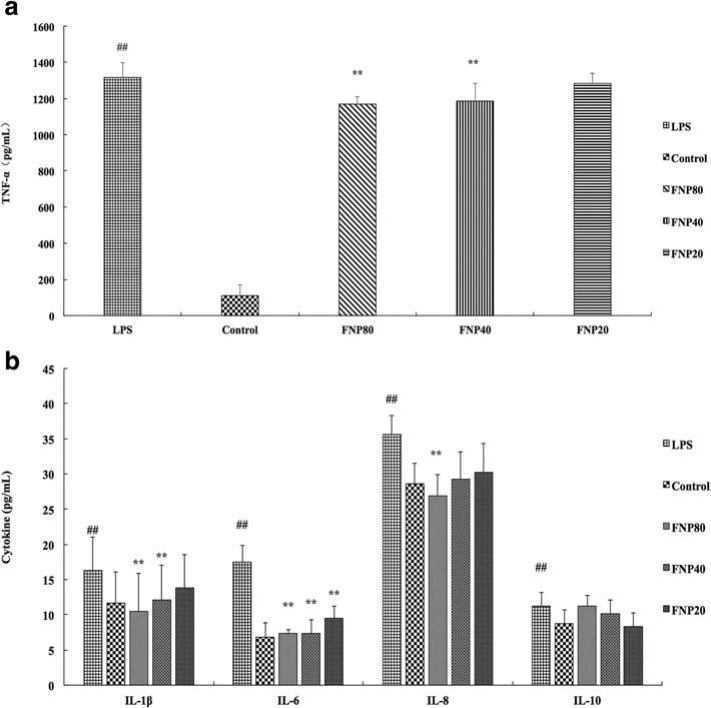
Effects of FNP on LPS-induced TNF-α (**a**) and IL-6, IL-8, IL-10 and IL-1β (**b**) productions in RAW264.7 macrophages. Notes: The cells were incubated for 24 h with 1 μg/mL of LPS in the absence or presence of FNP (20, 40, and 80 μg/mL). FNP was added 1 h before incubation with LPS. Inflammatory cytokine level were determined by cytokine-specific ELISA kit. Each column represented as the means ± SD from three independent experiments. _##_ *P* <0.01 vs control group; _*_ *P* <0.05 vs LPS group; _**_ *P* <0.01 vs LPS group

Inflammatory cytokine level were determined by cytokine-specific ELISA kit. RAW 264.7 cells with LPS treated alone resulted in significant increases in cytokine production (TNF-α, IL-1β, IL-6, IL-8 and IL-10) as compared to the control group (Fig. 6a and 6b). However, the production of IL-6 was significantly inhibited as pretreated with FNP (80, 40 and 20 μg/mL) (Fig. 6b). The production of TNF-α and IL-1β was significantly inhibited as pretreated with FNP (80 and 40 μg/mL) (Fig. 6a and 6b). Only pretreated by 80 μg/mL FNP significantly decreased production of IL-8 induced by LPS (Fig. 6b). FNP pretreatment showed no influence in production of IL-10 induced by LPS (Fig. 6b).

### Effects of FNP on iNOS and COX-2 protein expression in LPS-stimulated RAW 264.7 macrophages

**Fig. 7:**
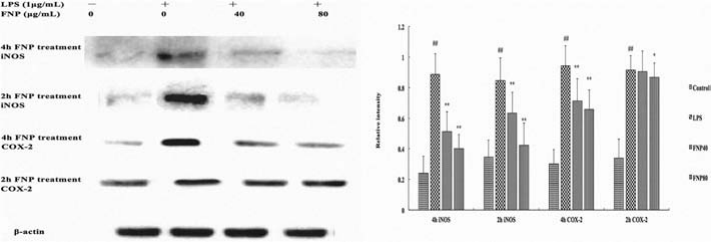
Effects of FNP on iNOS and COX-2 protein expression in LPS-stimulated RAW264.7 cells. Notes: The cells were pretreated with different concentrations (40 μg/mL, 80 μg/mL) of FNP for 2 h or 4 h and stimulated with 1 μg/mL of LPS for 24 h. Total cellular proteins (50 μg) were separated by SDS-PAGE, then transferred to PVDF membrane and detected by Western blot analysis. Quantification of iNOS and COX-2 protein expression was normalized to β-actin using a densitometer. Each column represented as the means ± SD from three independent experiments. _##_ *P* <0.01 vs control group; _*_ *P* <0.05 vs LPS group; _**_ *P* <0.01 vs LPS group

The expression levels of iNOS and COX-2 protein were examined by Western blot analysis using β-actin as an internal control. As shown in Fig. 7, RAW 264.7 cells with LPS treated alone resulted in significant increases in iNOS protein expressin, 4 h and 2 h FNP pretreatment (80 μg/mL) significantly inhibited iNOS protein expression induced by LPS, the iNOS protein expression induced by LPS was decreased by 50.2 % and 54.8 % respectively. The expression levels of COX-2 protein were strongly induced by LPS, pretreated with FNP (80 μg/mL) for 4 h significantly inhibited COX-2 protein expression induced by LPS the protein expression induced by LPS was decreased by 30.0 %.

### Effects of FNP on LPS-induced MAPKs and AMPK phosphorylation in LPS-stimulated RAW 264.7 macrophages

**Fig. 8:**
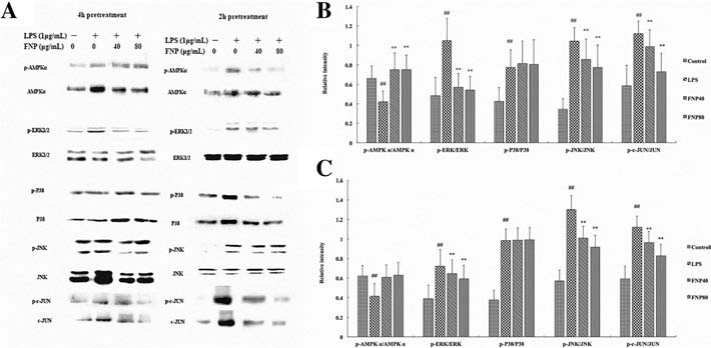
Effects of FNP on phosphorylation of MAPKs and AMPK in LPS-stimulated RAW264.7 cells. Notes: The cells were pretreated with different concentrations (40 μg/mL and 80 μg/mL) of FNP for 2 h or 4 h and stimulated with 1 μg/ml of LPS for 1 h. Total cellular proteins (50 μg) were separated by SDS-PAGE, then transferred to PVDF membrane and detected by Western blot analysis. Western blot results were shown in (**a**). Quantification of p-AMPKα, p-ERK1/2, p-P38, p-JNK and p-c-JUN protein expression was normalized to AMPKα, ERK1/2, P38, JNK and c-JUN using a densitometer. Each column represented as the means ± SD from three independent experiments. Results of 4h pretreatment were shown in (**b**) and results of 2 h pretreatment were shown in (**c**). _#_ *P* <0.05 vs control group; _##_P <0.01 vs control group; _*_ *P* <0.05 vs LPS group; _**_ *P* <0.01 vs LPS group

To investigate whether the inhibition of inflammatory mediator secretion by FNP is mediated through the MAPKs and AMPK pathway, we examined the effect of FNP on LPS-stimulated phosphorylation of ERK, JNK, p38 MAPKs,c-JUN and AMPKα in RAW 264.7 macrophages by Western blot analysis using phospho-specific antibodies. Cells were pretreated with different concentrationsof FNP for 2 h or 4 h and stimulated with 1 μg/ml of LPS for 1 h. As shown in Fig. 8b and 8c, pretreated with FNP (40 μg/mL and 80 μg/mL) for 2 h and 4 h resulted in the notably inhibition of LPS-induced phosphorylation of ERK, JNK and c-JUN, whereas it did not affect p38 phosphorylation. As shown in Fig. 8c, RAW 264.7 cells with LPS treated alone resulted in significant decreased in the ratio of p-AMPKα/ AMPKα, pretreated with FNP for 2h not affect the ratio of p-AMPKα/ AMPKα, whereas pretreated with FNP for 4 h notably increased it (Fig. 8b).

## Discussion

Catechin, epicatechin, hyperin, isoquercitrin, isorhamnetin, kaempferol, quercetin, quercitrin, rhamnazin and rutin was found to contain in Various extracts and fractions of *Polygonum hydropiper L.* whole plant and herbs [30–32]. In this study, the content of flavonoids was determined by spectrophotometry using the aluminium chloride colorimetric method. Rutin was used as a reference standard and the flavonoid content express in μg rutin/mL of FNP. The principle of aluminum chloride colorimetric method is that aluminum chloride forms acid stable complexes with the C-4 keto group and either the C-3 or C-5 hydroxyl group of flavones and flavonols [33]. Only rutin, quercetin and quercitrin were used as standards in in the HPLC identification, so contents of rutin, quercetin and quercitrin (50.7 %) were less than total flavonoid content in FNP (55.3 %). According to our HPLC results in chromatographic condition of acetonitrile and 0.3 % phosphoricacid (27:73, v/v), the contents of rutin, quercetin and quercitrin in FNP is 21.92 %, 8.22 % and 20.61 % respectively, the retention times is 7.92 min, 26.53 min and 11.91 min. According to the UPLC-MS identification in chromatographic condition of 19 % methanol and 0.1 % formic acid, the order of retention times of flavonoids in *Polygonum hydropiper L.* was found to be as follows: rutin < hyperin < isoquercitrin < quercitrin < catechin < epicatechin < quercetin < kaempferol < isorhamnetin [31]. We speculated FNP contain catechin and epicatechin.

Intraperitoneal injectetion of LPS has been used as a common experimental inflammation model and characterized by increased levels of neutrophils, protein content, inflammatory cytokines, chemokines and lung injury [26, 34]. In the present study, as expected, mice stimulated with LPS exhibited a high mortality. Pretreatment with FNP markedly reduced the mortalities indicated that FNP could significantly protect mice from LPS-induced death. Proinflammatory cytokines including TNF-α and IFN-γ, which playing a prominent role in the pathogenesis of tissue injury evolving from septic shock, are contributing to the protection of the lung against severe damage [35]. In a Gram-positive sepsis modle, mRNA expression levels of TNF-α and IFN-γ significantly increase [36]. Next, we investigated the inhibitory effect of FNP on histological alterations and mRNA expression levels of proinflammatory cytokines in lung. In our study,FNP pretreatment dramatic decreased the mRNA expression of TNF-α, IFN-α, IFN-γ and IL-2 induced by LPS in lung, attenuated lung injury by decreasing alveolar incrassation and PMN infiltration. These results showed that FNP exert a salutary effect on acute lung injury in LPS-stimulated mice. Reactive oxygen species have been found to have a role in the pathogenesis of LPS-induced gastrointestinal motility disturbances [37]. LPS injection increases the levels of oxidative damage in plasma and intestine [38]. We investigated the inhibitory effect of FNP on MDA and MPO levels in small intestine homogenates, histological alterations in ileum and colon. Our results suggested that FNP pretreatment attenuated ileum and colon injuries by decreasing MDA and MPO levels, these results showed that FNP exert a salutary effect on intestinal damage in LPS-stimulated mice. Thus, we can draw a conclusion that FNP exerted benefits on lung and intestinal injury in LPS-stimulated mice.

The pathogenesis of sepsis involves disorders of oxidant/ anti-oxidant and inflammation/anti-inflammation, and the increased production of inflammatory cytokines. ROS, NO and the potent proinflammatory cytokines (such as TNF-α, IL-1β and IL-6) play an important role in the occurrence and development of systemic inflammatory responses [39, 40]. Flavonoid compound derived from natural plant inhibits the activation of macrophage and protects mice from macrophage-mediated endotoxin shock [41]. In the present study, the results of in vivo experiment showed that FNP pretreatment suppressed LPS-induced TNF-α production in serum, liver and small intestine. In addition, the results of in vitro experiment showed that FNP pretreatment suppressed LPS-induced inflammatory cytokines (TNF-α, IL-1β、IL-6、IL-8), NO and ROS production in RAW 264.7 cells. Considering a critical role of ROS, NO and inflammatory cytokines in septic shock, increased survival of mice and reduction of multiple organ injury might be mediated by inhibition the production of ROS, NO and inflammatory cytokines in activated macrophage.

To alleviate cumulative burden of oxidative stress, cells generally utilize anti-oxidant defense systems to scavenge ROS. SOD and GSH-PX are the first line of defense against oxidative stress and can inhibit free radical formation and prevent oxidative damage by ROS [42]. In this paper, to determine whether the protective effects of FNP on multiple organ injury in LPS-stimulated mice were mediated by their antioxidant functions, the activities of antioxidant enzymes in mice under sepsis with or without pretreatment of FNP were investigated. In the present study, the results of in vivo experiment showed that FNP pretreatment significantly promoted T-AOC, T-SOD, GSH-PX and GSH levels as compared to LPS group, suggesting that FNP pretreatment significantly attenuated the oxidative damage of LPS-stimulated mice by maintaining the activity of antioxidant enzymes.

Over production of NO and PGE_2_ was due to overexpression of iNOS and COX-2. During infection, inflammation injury was caused by uncontrolled release of NO, while PGE_2_ was contributed to edema, angiogenesis and invasion [43]. To explore possible mechanism of anti-inflammatory effect by FNP pretreatment, we study its effects on iNOS and COX-2 protein expression in LPS-stimulated RAW264.7 cells. In the present study, our results showed that FNP pretreatment significantly attenuated the LPS-induced up-regulation of iNOS, COX-2 protein expressions in RAW264.7 macrophages, indicating that the inhibition of NO and PGE2 production by FNP is a result of the inhibition of iNOS and COX-2 protein expressions. AMPK activation suppressed the LPS-induced expression of pro-inflammatory cytokines in glial cells [44], vascular endothelial cells [45] and BV-2 microglia cells [46]. However, AMPK activation promoted inflammatory cytokines production in in cardiac fibroblasts of adult mice [47] or human synovial fibroblast cells [48]. To verify the role of AMPKα in macrophage, we investigated the phosphorylation of p-AMPKα/ AMPKα in LPS-induced RAW264.7 cells and observed a decrease in ratio of p-AMPKα/AMPKα. FNP pretreatment increase ratio of p-AMPKα/AMPKα, suggesting that the anti-inflammatory activity of FNP was partly regulated by AMPKα1 subunit.

ERK, JNK and p38, the activation and phosphorylation of these three subtypes MAPKs play certain roles in anti-inflammatory mechanism in LPS-induced macrophages [49]. Evidence were shown that p38 and ERK are involved in iNOS production in LPS-induced RAW264.7 cells [50], while another study showed that p38 and ERK activation up-regulates LPS-induced COX-2 expression in RAW264.7 cells [51]. In the present study, our results observed that FNP pretreatment significantly inhibited the phosphorylation/activation of JNK and ERK in LPS-induced RAW264.7 macrophages, while extended pretreatment to 4 h, stronger inhibiting effect in these subtypes MAPKs were shown. These results suggesting suppression of FNP pretreatment on inflammatory mediators may be regulated by the ERK and JNK MAPKs signaling pathways. Moreover, activated JNK induced phosphorylation of c-JUN in ser73 and ser63. Our results indicated that FNP pretreatment suppressed phosphorylation/activation of c-JUN induced by LPS. Thus, we can draw a conclusion that anti-inflammatory activities of FNP may be mediated by inhibiting the phosphorylation of JNK, ERK and c-JUN in MAPKs signaling pathways.

## Conclusions

Our present study demonstrated that FNP isolated from *Polygonum hydropiper L.* possesses antioxidant and anti-inflammatory activities. The anti-inflammatory mechanism of FNP might be related to the decrement of the level of MDA, MPO and ACP via increasing the activities of T-AOC, T-SOD, GSH-PX and GSH. FNP inhibited iNOS and COX-2 protein expression in LPS-stimulated RAW264.7 macrophages. The inhibitory effects of FNP may be mediated by suppression of JNK, ERK MAPKs signaling pathways.

## Abbreviations

ACP: Acid phosphatase
AMPK: AMP-activated protein kinase
BSA: Bovine serum albumin
COX-2: Cyclooxygenase-2
CT value: Cycle threshold
DCFH-DA: Dichlorofluorescin diacetate
DMEM: Dulbecco minimum essential medium
DMSO: Dimethyl Sulphoxide
ECL: Enhanced chemiluminescence
ELISA: Enzyme-linked immuno sorbent assay
ERK: Extracellular signal regulated kinase
FNP: Flavonoids from normal butanol fraction of *Polygonum hydropiper L.* extract
GSH: Glutathione
GSH-PX: Glutathione peroxidase
HPLC: High Performance Liquid Chromatography
IFN-α: Interferon-α
IFN-γ: Interferon-γ
IL-10: Interleukin-10
IL-1β: Interleukin-1β
IL-6: Interleukin-6
IL-8: Interleukin-8
iNOS: inducible nitric oxide synthase
JNK: c-Jun N terminal kinase
LPS: Lipopolysaccharide
LZM: Lysozyme
MAPKs: Mitogen-activated protein kinases
MDA: Malondialdehyde
MPO: Myeloperoxidase
mRNA: messenger RNA
MTT: 3-(4, 5-Dimethylthiazol-2-yl)-2, 5-diphenyltetrazolium bromide
NO: Nitric oxide
NOS: Nitric oxide synthase
PBS: Phosphate-buffered saline
PGC-1α: Peroxisome proliferator-activated receptor-γ coactivator 1 α
PGE2: Prostaglandin E2
PMN: Polymorphonuclear neutrophil
RNS: Reactive nitrogen species
ROS: Reactive oxygen species
T-AOC: Total antioxidant capacity
TNF-α: Tumor necrosis factor-α
T-SOD: Total superoxidase dismutase
TTBS: Tween 20/ Tris buffered saline
